# Fournier’s gangrene with anal cancer: a case report and literature review

**DOI:** 10.1093/jscr/rjaf573

**Published:** 2025-08-12

**Authors:** Yunmin Lee, Kyeongeui Kim, Chanhee Park, Jeongwoo Lee, Sunguk Bae, Seongkyu Baek, Woonkyung Jeong

**Affiliations:** Department of Surgery, Keimyung University Dongsan Medical Center, 1035 Dalgubeol-daero, Dalseo-gu, Daegu, 42601, Republic of Korea; Department of Surgery, Armed Forces Yangju Hospital, 483 Pyeonghwa-ro, Yangju-si, Gyeonggi-do, 11430, Republic of Korea; Department of Surgery, Keimyung University Dongsan Medical Center, 1035 Dalgubeol-daero, Dalseo-gu, Daegu, 42601, Republic of Korea; Department of Surgery, Keimyung University Dongsan Medical Center, 1035 Dalgubeol-daero, Dalseo-gu, Daegu, 42601, Republic of Korea; Department of Surgery, Keimyung University Dongsan Medical Center, 1035 Dalgubeol-daero, Dalseo-gu, Daegu, 42601, Republic of Korea; Department of Surgery, Keimyung University Dongsan Medical Center, 1035 Dalgubeol-daero, Dalseo-gu, Daegu, 42601, Republic of Korea; Department of Surgery, Keimyung University Dongsan Medical Center, 1035 Dalgubeol-daero, Dalseo-gu, Daegu, 42601, Republic of Korea

**Keywords:** Fournier gangrene, soft tissue infections, anus neoplasms, chemoradiotherapy, colorectal surgery

## Abstract

Fournier’s gangrene (FG) is a rapidly progressing necrotizing fasciitis affecting the perineum and perianal area, often necessitating urgent surgical intervention. While commonly associated with diabetes, immunosuppression, and infections, malignancy-related FG is uncommon. A 59-year-old female presented with severe left buttock pain and perianal erythema. Imaging studies revealed perineal abscess formation and severe emphysema, leading to a diagnosis of FG. Despite initial surgical debridement and intensive care, persistent perianal induration prompted a biopsy, which confirmed squamous cell carcinoma with HPV 16 positivity. Chemoradiotherapy was administered, achieving significant regression of the tumor and lymph nodes. Robotic abdominoperineal resection was subsequently performed, achieving complete tumor regression (ypT0N0). The patient recovered well initially, but systemic recurrence occurred 10 months after surgery. This case illustrates the infrequent coexistence of FG and anal cancer, highlighting the necessity for early malignancy evaluation in patients with FG.

## Introduction

Fournier’s gangrene (FG), a relatively rare form of necrotizing fasciitis, aggressively progresses, affecting the superficial and deep tissues of the perineum, perianal, scrotal, and genital areas [1]. FG frequently originates from infections or trauma in the genitourinary and anorectal tracts or perineum. This life-threatening condition necessitates immediate surgical intervention, broad-spectrum antibiotics, and intensive supportive care [2, 3].

Anal cancer, a relatively uncommon malignancy, represents ~2%–4% of all gastrointestinal malignancies, with squamous cell carcinoma as the most prevalent histologic subtype [4]. Diagnosis of anal cancer is often delayed as its symptoms resemble benign conditions, and coexisting FG can further obscure tumor progression by mimicking inflammation [5].

This report presents a case of anal cancer-induced FG and reviews the literature to outline optimal diagnostic and treatment strategies for this rare condition.

## Case report

**Figure 1:**
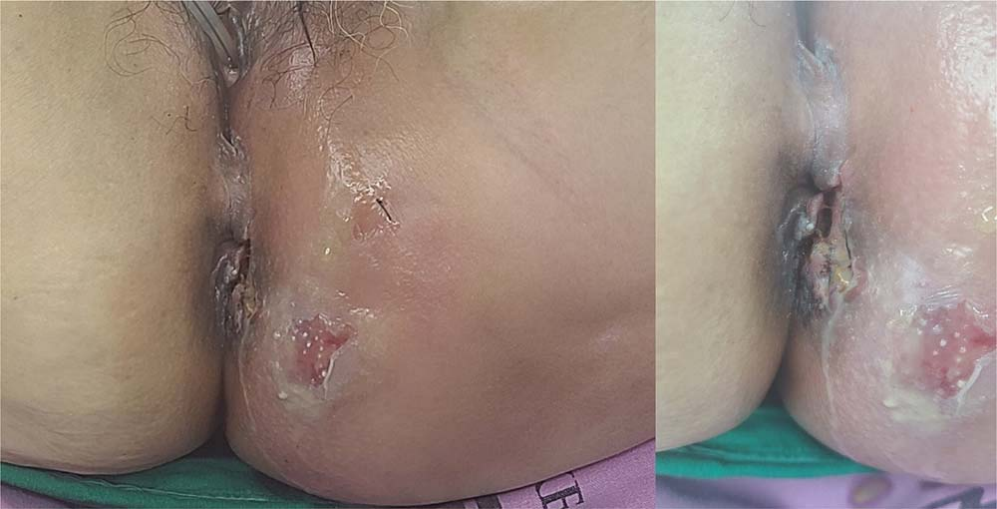
Patient images of Fournier’s gangrene with multifocal necrotizing fasciitis.

**Figure 2:**
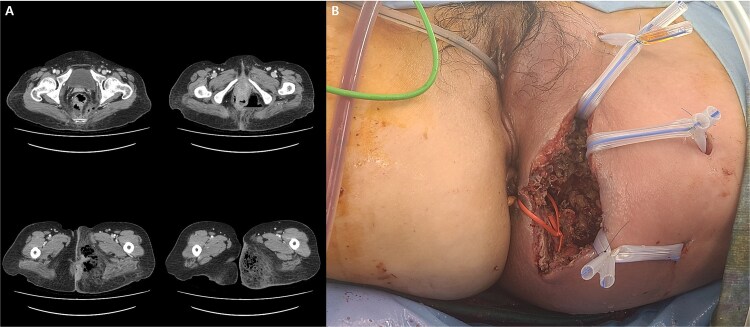
(A) Abscess formation with emphysema in the left perineum and buttock on abdominal pelvic CT scan. (B) Postoperative images after incision and drainage.

**Figure 3:**
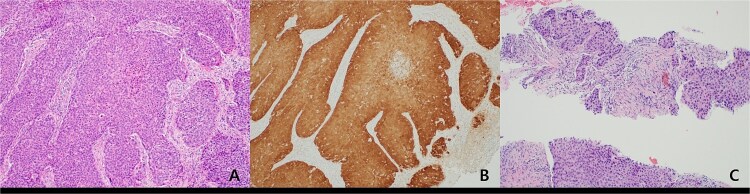
(A) Histopathologic features of anal cancer showing infiltrating non-keratinizing squamous cell carcinoma. (B) Diffuse and strong ‘block-type’ p16 immunoreactivity is observed in the tumor nests. This finding was also confirmed by PCR for HPV type 16. (C) Metastatic squamous cell carcinoma showing histologic features similar to the primary anal cancer is identified in a supraclavicular lymph node (original magnification, ×100).

**Figure 4:**
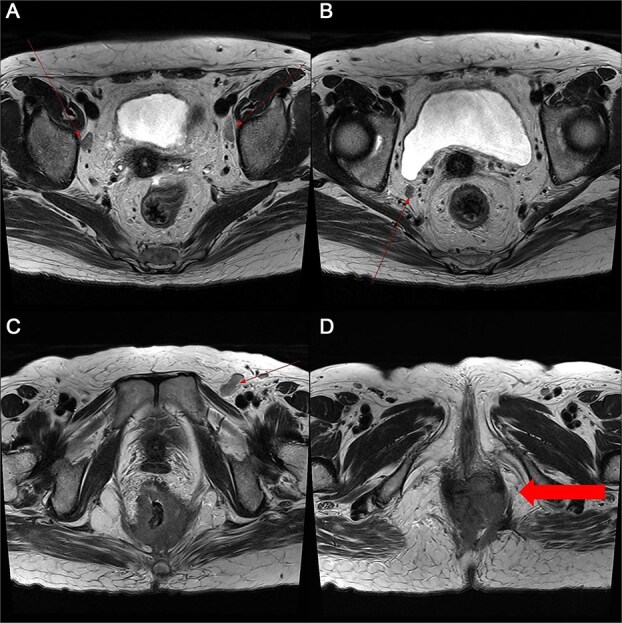
Pelvic MRI demonstrating the progression and extent of perineum and pelvic involvement. (A–C) Indeterminate lymph nodes in the bilateral external iliac chains, right obturator chain, and bilateral inguinal chains (thin arrow). (D) Presence of anorectal cancer with invasion of both levator muscles (thick arrow).

**Figure 5:**
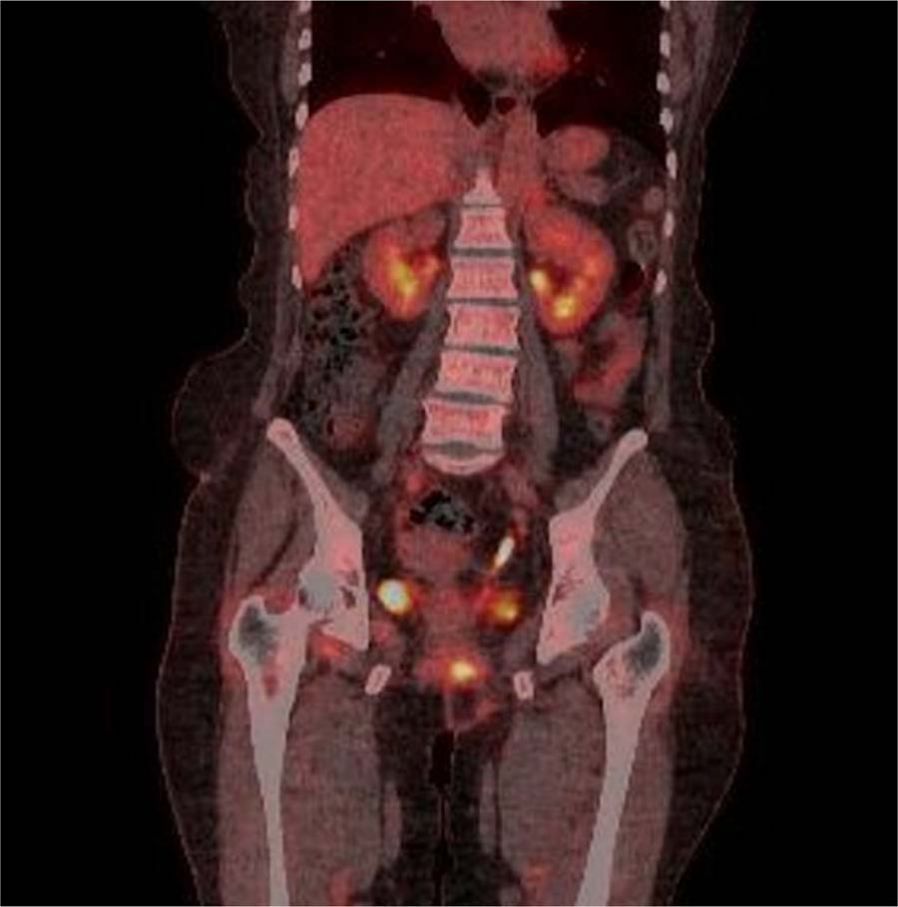
Multiple intense hypermetabolic mass lesions identified from the anus to the rectum, accompanied by numerous hypermetabolic lymph nodes in the pelvis.

**Figure 6:**
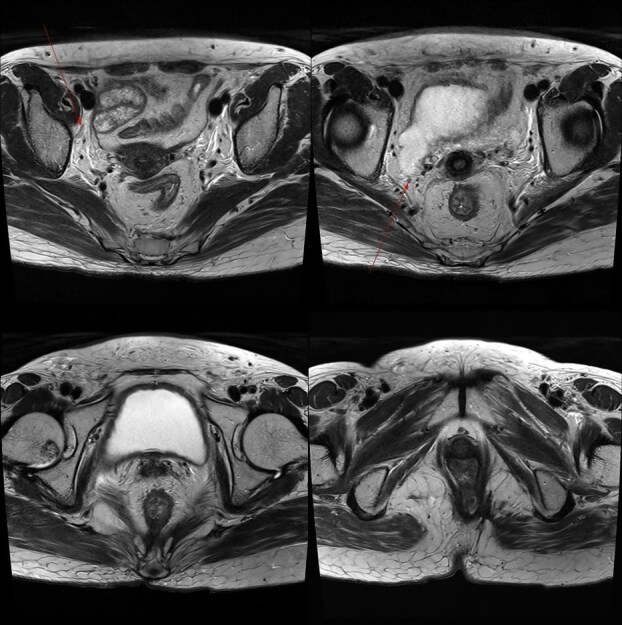
Marked reduction in the size of the tumor and lymph nodes following CCRT.

A 59-year-old female patient was referred to our hospital with severe left buttock pain lasting 5 days. Although she had no co-morbidities, she was previously diagnosed with hemorrhoids at the local clinic due to perianal pain persisting for 1 month. The patient had no medical or surgical history. Her initial body temperature was 38.4°C, blood pressure was 110/60 mmHg, heart rate was 98 beats/min, and her oxygen saturation was 98% on room air. On physical examination, erythema and hardness were noted in the left perineum and buttock areas. Additionally, dilated piles were observed on anus (Fig. 1). Given the clinical suspicion of FG, a computed tomography (CT) scan and blood tests were urgently performed. The CT scan demonstrated abscess formation along with severe emphysema in the left perineum and buttock (Fig. 2A). Blood tests showed leukocytosis of 41 840/μl with neutrophil left shift and an elevated C-reactive protein level of 33.4 ng/dl. The patient was finally diagnosed with FG, indicated by a severity index score of 6 points [6]. After initiating fluid resuscitation and broad-spectrum antibiotics, extensive debridement of the perineum was executed (Fig. 2B). However, due to clinical deterioration after general anesthesia, staged debridement was performed, and the patient was admitted to the intensive care unit for stabilization. Subsequent debridement and a diverting colostomy were performed 3 days after surgery. After 1 week in intensive care, the patient was transferred to the general ward where additional wound debridement and revisions were conducted. Two months after initial surgery, the surgical wound had completely healed, and wound closure was performed. Although inflammation had subsided, indurated tissue persisted around the anus, which had become so constricted that a digital rectal examination was impeded. Therefore, a biopsy of the perianal tissue was taken. Histopathological examination identified moderately differentiated squamous cell carcinoma, and HPV 16 was detected (Fig. 3). Pelvic magnetic resonance imaging (MRI) revealed anorectal cancer with invasion into both levator muscles and indeterminate lymph nodes in the bilateral external iliac, right obturator, and bilateral inguinal chains (Fig. 4). Positron emission tomography demonstrated an intensely hypermetabolic mass extending from the anus to the rectum, accompanied by multiple hypermetabolic lymph nodes in the left common iliac, left external iliac, right internal iliac, and bilateral inguinal regions (Fig. 5). Consequently, concurrent chemoradiotherapy (CCRT) employing mitomycin and 5-fluorouracil was initiated. The patient received a total radiation dose of 63 Gy in 35 fractions over 8 weeks. Elective nodal irradiation included the bilateral inguinal, internal iliac, and mesorectal nodal basins, in accordance with standard guidelines. Although follow-up MRI after CCRT showed a significant reduction in the size of the primary tumor and lymph nodes, we decided to perform a radical resection (Fig. 6). Robotic abdominoperineal resection was performed 3 months after CCRT, during which lateral pelvic lymph node dissection wasn’t conducted, as post CCRT imaging showed indeterminate lymph nodes suggestive of remission. Histopathological examination of the specimen revealed no residual tumor (ypT0N0). Although a minor surgical site infection occurred at the perineal wound, it was successfully treated with oral antibiotics and dressings at an outpatient setting. At 10 months post-surgery, the patient reported left pelvic pain, and follow-up imaging revealed suspected metastases in the left psoas, para-aortic area, supraclavicular node, right 10th rib, and lung. Consequently, the patient was scheduled for palliative chemotherapy.

## Discussion

FG is most commonly associated with diabetes, immunosuppression, and perineal infections, though its occurrence secondary to malignancy is exceedingly rare [7–10]. To the best of our knowledge, this is the first case report of FG occurring with anal cancer. In this case, the underlying anal squamous cell carcinoma was initially obscured by the severe infectious process, leading to a diagnostic delay. The progression of FG against a backdrop of undiagnosed malignancy underscores the need for comprehensive evaluation in cases with ambiguous etiology, especially in patients without typical risk factors.

The relationship between FG and malignancy remains unclear, with potential mechanisms including tumor-related immunosuppression, local tissue invasion, and superimposed infections due to compromised tissue integrity [11, 12]. We hypothesized that FG may have contributed to anal cancer development via chronic ulceration, infection, and immune dysfunction, alongside HPV 16 infection as a known high-risk factor. This suggests that FG could be an unusual initial presentation of anorectal malignancies, necessitating a high index of suspicion among patients with atypical disease courses.

The role of CCRT in treating anal cancer is well-established, as it demonstrates efficacy in tumor downstaging for definitive treatment and contributes to improved surgical outcomes [13]. However, given the rarity of anal cancer–induced FG, guidelines on the use and timing of CCRT are lacking, and its immunosuppressive effects may further increase the risk of tissue necrosis and FG. This raises concerns about its impact on infection control and the risk of FG recurrence in oncologic patients. Given these conflicting views, meticulous patient selection and vigilant monitoring are imperative when administering CCRT in cases with active or previous FG.

Anal cancer that responds to CCRT generally exhibits a favorable prognosis [14]. In this case, definitive surgery was performed despite substantial tumor regression after CCRT to achieve complete oncologic control. This decision was driven by residual tissue changes, the high risk of lingering malignancy despite imaging results, and the necessity for definitive resection to prevent recurrence. Although the final pathology (ypT0N0) confirmed complete tumor eradication, the detection of metastases 10 months later reflected the disease’s aggressive nature. This case highlights that anal cancer associated with Fournier’s gangrene can exhibit aggressive clinical behavior despite an initial complete response to CCRT, underscoring the importance of meticulous and structured follow-up. Given the risk of delayed distant metastasis, intensified surveillance, including physical examination and imaging every 3 months during the first year, every 6 months in the second year, and annually thereafter, is warranted. This approach might help in the early detection of recurrence and inform timely therapeutic interventions. FG rarely accompanies malignancy, particularly anal cancer, but in patients without typical risk factors, imaging and biopsy are essential to exclude underlying tumors. Moreover, the presence of an underlying malignancy could suggest aggressive tumor biology, necessitating urgent and definitive oncologic management.
